# Estimating aboveground net biomass change for tropical and subtropical forests: Refinement of IPCC default rates using forest plot data

**DOI:** 10.1111/gcb.14767

**Published:** 2019-08-16

**Authors:** Daniela Requena Suarez, Danaë M. A. Rozendaal, Veronique De Sy, Oliver L. Phillips, Esteban Alvarez‐Dávila, Kristina Anderson‐Teixeira, Alejandro Araujo‐Murakami, Luzmila Arroyo, Timothy R. Baker, Frans Bongers, Roel J. W. Brienen, Sarah Carter, Susan C. Cook‐Patton, Ted R. Feldpausch, Bronson W. Griscom, Nancy Harris, Bruno Hérault, Eurídice N. Honorio Coronado, Sara M. Leavitt, Simon L. Lewis, Beatriz S. Marimon, Abel Monteagudo Mendoza, Justin Kassi N'dja, Anny Estelle N'Guessan, Lourens Poorter, Lan Qie, Ervan Rutishauser, Plinio Sist, Bonaventure Sonké, Martin J. P. Sullivan, Emilio Vilanova, Maria M. H. Wang, Christopher Martius, Martin Herold

**Affiliations:** ^1^ Laboratory of Geo‐Information Science and Remote Sensing Wageningen University and Research Wageningen The Netherlands; ^2^ Plant Production Systems Group Wageningen University and Research Wageningen The Netherlands; ^3^ Centre for Crop Systems Analysis Wageningen University and Research Wageningen The Netherlands; ^4^ School of Geography University of Leeds Leeds UK; ^5^ Escuela de Ciencias agrícolas, pecuarias y ambientales Universidad Nacional Abierta y a Distancia Bogota Colombia; ^6^ Fundación ConVida Medellín Colombia; ^7^ Conservation Ecology Center Smithsonian Conservation Biology Institute Front Royal VR USA; ^8^ Center for Tropical Forest Science‐Forest Global Earth Observatory Smithsonian Tropical Research Institute Panama Republic of Panama; ^9^ Museo de Historia Natural Noel Kempff Mercado Universidad Autónoma Gabriel René Moreno Santa Cruz Bolivia; ^10^ Universidad Autónoma Gabriel René Moreno Santa Cruz Bolivia; ^11^ Forest Ecology and Forest Management Group Wageningen University and Research Wageningen The Netherlands; ^12^ The Nature Conservancy Arlington VR USA; ^13^ Geography College of Life and Environmental Sciences University of Exeter Exeter UK; ^14^ World Resources Institute Washington DC USA; ^15^ CIRAD, UR Forests & Societies University of Montpellier Montpellier France; ^16^ Institut National Polytechnique Félix Houphouet‐Boigny Yamoussoukro Ivory Coast; ^17^ Instituto de Investigaciones de la Amazonía Peruana Iquitos Peru; ^18^ Department of Geography University College London London UK; ^19^ Campus de Nova Xavantina Universidade do Estado de Mato Grosso Nova Xavantina Brazil; ^20^ Jardín Botánico de Missouri Oxapampa Peru; ^21^ Universidad Nacional de San Antonio Abad del Cusco Cusco Peru; ^22^ UFR Biosciences Laboratoire de Botanique Université Félix Houphouet‐Boigny Abidjan Ivory Coast; ^23^ School of Life Sciences University of Lincoln Lincoln UK; ^24^ Plant Systematic and Ecology Laboratory University of Yaoundé Yaoundé Cameroon; ^25^ Universidad de Los Andes Mérida Venezuela; ^26^ School of Environmental and Forest Sciences University of Washington Seattle WA USA; ^27^ Department of Animal & Plant Sciences University of Sheffield Sheffield UK; ^28^ Center for International Forestry Research (CIFOR) Bogor Indonesia

**Keywords:** biomass change, global ecological zones, IPCC, managed and logged forests, old‐growth forests, secondary forests, (sub)tropical forests

## Abstract

As countries advance in greenhouse gas (GHG) accounting for climate change mitigation, consistent estimates of aboveground net biomass change (∆AGB) are needed. Countries with limited forest monitoring capabilities in the tropics and subtropics rely on IPCC 2006 default ∆AGB rates, which are values per ecological zone, per continent. Similarly, research into forest biomass change at a large scale also makes use of these rates. IPCC 2006 default rates come from a handful of studies, provide no uncertainty indications and do not distinguish between older secondary forests and old‐growth forests. As part of the 2019 Refinement to the 2006 IPCC Guidelines for National Greenhouse Gas Inventories, we incorporate ∆AGB data available from 2006 onwards, comprising 176 chronosequences in secondary forests and 536 permanent plots in old‐growth and managed/logged forests located in 42 countries in Africa, North and South America and Asia. We generated ∆AGB rate estimates for younger secondary forests (≤20 years), older secondary forests (>20 years and up to 100 years) and old‐growth forests, and accounted for uncertainties in our estimates. In tropical rainforests, for which data availability was the highest, our ∆AGB rate estimates ranged from 3.4 (Asia) to 7.6 (Africa) Mg ha^−1^ year^−1^ in younger secondary forests, from 2.3 (North and South America) to 3.5 (Africa) Mg ha^−1^ year^−1^ in older secondary forests, and 0.7 (Asia) to 1.3 (Africa) Mg ha^−1^ year^−1^ in old‐growth forests. We provide a rigorous and traceable refinement of the IPCC 2006 default rates in tropical and subtropical ecological zones, and identify which areas require more research on ∆AGB. In this respect, this study should be considered as an important step towards quantifying the role of tropical and subtropical forests as carbon sinks with higher accuracy; our new rates can be used for large‐scale GHG accounting by governmental bodies, nongovernmental organizations and in scientific research.

## Graphical abstract

As countries advance in greenhouse gas (GHG) accounting for climate change mitigation, consistent estimates of aboveground biomass change (∆AGB) in natural forests are needed. Countries with limited forest monitoring capabilities in the (sub)tropics rely on the 2006 IPCC guidelines for default ∆AGB rates. This study provides refined default ∆AGB rate estimates based on forest plot data in younger secondary forests, older secondary forests and old‐growth forests, located in 42 countries across (sub)tropical ecozones. These new estimates contribute towards the 2019 IPCC Refinement and can be used for large‐scale GHG accounting by governmental bodies, nongovernmental organizations and in scientific research.

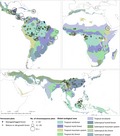

## 1 INTRODUCTION

Signatory nations of the Paris Agreement agreed to report on greenhouse gas (GHG) emissions and removals for climate change mitigation efforts (UNFCCC, 2015). Reporting requires providing the UNFCCC with reliable estimates of anthropogenic CO_2_ emissions based on anthropogenic activity data and removals based on ecosystem‐level GHG fluxes. In this respect, forest ecosystems are a central terrestrial component of the global carbon (C) cycle, storing roughly half of terrestrial C (Bonan, 2008) and generally acting as C sinks (Houghton, 2007). Tropical and subtropical forests account for approximately 70% of the world's gross forest C sink (Pan et al., 2011), and through their conservation and restoration, they have the potential to partially offset CO_2_ anthropogenic emissions (Houghton, Byers, & Nassikas, 2015). Thus, accounting for GHG removals from the atmosphere through tropical and subtropical forest C sinks is of utmost importance.

Countries with tropical and subtropical forests can benefit from climate change mitigation policies through land restoration initiatives and Reducing Emissions from Deforestation and Forest Degradation (REDD+) schemes as a way to conserve and enhance their forest C sinks. These initiatives and schemes require monitoring, reporting and verification systems to account for forest C pools and fluxes (Turnhout et al., 2017) and should follow IPCC good practice guidelines (IPCC, 2003, 2006).

Due to the complexity of these ecosystems, as well as the often limited national forest monitoring capacities within the tropics, there are scarce country‐specific data on C sinks in natural forests. Thus, tropical countries rely heavily on default values (Tier 1) specified in the IPCC guidelines (IPCC, 2006), rather than using country‐specific data (Tier 2) or higher level methods such as repeated measurements in permanent plots (Tier 3). For example, for forest C pool reporting of tropical countries by 2015, 84 out of 99 countries were reporting at only Tier 1 level (Romijn et al., 2015).

IPCC 2006 Tier 1 forest C pools and sinks in natural forests are characterized in part as aboveground live tree biomass (AGB) and rates of aboveground net biomass change (∆AGB). In this context, ∆AGB is defined as the balance between annual rates of AGB gain (productivity and recruitment) and loss (mortality) over time and per unit area. IPCC 2006 Tier 1 default ∆AGB rates consist of single values and/or ranges (IPCC, 2006, Table 4.9) which provide spatially coarse estimates of ∆AGB across global ecological zones (FAO, 2012). Besides being widely used by countries for C reporting (FAO, 2015a, 2015b Appendix 4; Romijn et al., 2015), these default rates are also commonly used in research on forest biomass change and forest C fluxes (Achard et al., 2014; Viglizzo et al., 2011). To provide a thorough characterization of natural forest C sinks, IPCC 2006 default ∆AGB rates can be used together with other Tier 1 default values—such as AGB, belowground biomass (BGB) to AGB ratios—and loss estimates of AGB by anthropogenic activities. Natural forest C sink estimates are used alongside planted forest C sink estimates, which can then be combined with spatially explicit information such as forest cover and its change over time, as well as land‐use maps, to provide globally consistent estimates of AGB and BGB (e.g. Ruesch & Gibbs, 2008) and of changes in forest C pools (e.g. Petrescu, Abad‐Viñas, Janssens‐Maenhout, Blujdea, & Grassi, 2012).

While influential, IPCC 2006 default ∆AGB rates require improvement, since they incorporate only a fraction of the currently available forest plot data. Since the first compilation of these rates, new and expanding databases have greatly enlarged the amount of readily available and high‐quality tropical and subtropical forest plot data (Anderson‐Teixeira, Wang, McGarvey, & LeBauer, 2016). In addition, the IPCC 2006 default tables do not provide measures of variation, which is why the uncertainty of estimates cannot be characterized based on their values. Furthermore, there is no information on how these values were selected or derived, or how representative they are of the forests they describe.

Finally, IPCC 2006 default ∆AGB rates divide natural forest stands into above and below 20 years, which is a broad classification that does not account for known age‐related variation in secondary forests—which are naturally regenerated forest stands that regrow after natural or anthropogenic disturbances. Younger secondary forests have high ∆AGB rates (Anderson‐Teixeira et al., 2016; Poorter et al., 2016), which then decrease over the course of forest succession (Chazdon et al., 2007; Houghton, 2005) until the stand reaches a mature (further referred to as *old‐growth*) state in which ∆AGB slows down. Old‐growth forests may locally fluctuate between AGB gains and losses over time (Brienen et al., 2015; Chambers et al., 2013; Phillips et al., 1998), but most old‐growth tropical forest has on average contributed a net sink (e.g. Espírito‐Santo et al., 2014; Lewis et al., 2009; Pan et al., 2011). Since ∆AGB rates are expected to vary over the course of succession, secondary forests over 20 years should be disaggregated from old‐growth forest stands.

Managed and/or logged forests can also have high ∆AGB rates, since timber extraction and silvicultural treatments partially open the forest canopy, increasing the ∆AGB rate in the remaining stand (Rutishauser et al., 2015). Until recently, managed/logged forests have been largely overlooked when quantifying the contribution of tropical and subtropical forests to the global terrestrial C sink, even though they represent approximately 20% of the world's humid tropical forests (Asner, Rudel, Aide, Defries, & Emerson, 2009).

In this study, as part of the 2019 Refinement to the 2006 IPCC Guidelines for National Greenhouse Gas Inventories (IPCC, 2019), we refine the IPCC 2006 default ∆AGB rates in tropical and subtropical ecological zones. In the interest of facilitating the scientific use and future update of these default rates, we (a) incorporate newly available data on secondary, old‐growth and managed/logged forests; (b) disaggregate forests over 20 years into older secondary and old‐growth forests; (c) derive ∆AGB rate estimates in a clear, rigorous and reproducible manner; and (d) identify areas where better ∆AGB data are needed.

## 2 MATERIALS AND METHODS

### 2.1 Data compilation

We compiled AGB (Mg/ha; linked with stand age) and ∆AGB (Mg ha^−1^ year^−1^) data from existing plot networks, databases and primary scientific literature on natural, as opposed to planted, forest stands (Anderson‐Teixeira, Hermman, et al., 2018; Anderson‐Teixeira, Wang, et al., 2018; Anderson‐Teixeira et al., 2016; Brienen et al., 2015; Cook‐Patton et al., under review; Lewis et al., 2009; Poorter et al., 2016; Qie et al., 2017; Rutishauser et al., 2015) in global tropical and subtropical ecological zones (hereafter referred to as *ecozones*) as defined by FAO (2012). Additional studies not present in these databases were obtained through a review of studies in the Web of Science (v.5.26.2). Data were only included if they were present in a peer‐reviewed source, within the main text, as part of a table or as supplementary material. All data had to be georeferenced for aggregation by continent (North and South America, Africa or Asia) and ecozone. Following IPCC guidelines, studies with sites in continental United States were excluded from this compilation.

We divided forest plot data based on stand age or the presence of anthropogenic intervention. Following the methodology by Poorter et al. (2016), we included data from secondary forests stands up until 100 years. These data were then divided into younger secondary forests (≤20 years; as per the IPCC 2006 values) and older secondary forests (>20 years), based on their stand age or on the time since the last anthropogenic disturbance. Forest stands with no record of anthropogenic disturbance for at least the past 100 years were regarded as old‐growth forests. Forest stands with anthropogenic interventions resulting in partial stand disturbance such as silvicultural treatments or selective logging were regarded as managed/logged forests (Sist et al., 2015).

In old‐growth and managed/logged forests, ∆AGB is monitored mainly through repeated measurements of permanent plots (Brienen et al., 2015; Chave et al., 2008; Lewis et al., 2009; Muller‐Landau, Detto, Chisholm, Hubbell, & Condit, 2014; Qie et al., 2017; Sist et al., 2015), while the study of ∆AGB in secondary forests relies mostly on chronosequences (Chazdon et al., 2007, 2016; Poorter et al., 2016). A chronosequence consists of static measurements (i.e. AGB) of plots under similar environmental conditions that differ in their age since abandonment. Chronosequences use, therefore, a space‐for‐time substitution to estimate long‐term successional change without monitoring individual plots over long time periods, and provide a critical data source given the large practical challenges to monitoring recovering forests for many decades.

Secondary forest chronosequences consisted of AGB or C (Mg/ha) plots at different stand ages per chronosequence site. For North and South America, only chronosequences with ≥3 chronosequence plots were included to generate site‐specific AGB–stand age relationships. For Asia and Africa, where fewer data were available, proximate sites (<1.5° in Africa and <4.0° in Asia) in the same ecozone were grouped and treated like single chronosequences. This permitted us to include data from 18 sites in Asia and nine sites in Africa which contained only one or two plots each.

For old‐growth forests and managed/logged forests, we included ∆AGB (or ∆C) rates from permanent plots. For ∆AGB rates (Mg ha^−1^ year^−1^), each plot had at least one ∆AGB value based on two consecutive measurements (one census interval) of the same plot. When aboveground C (or aboveground ∆C) was reported, we converted these values to AGB or ∆AGB by dividing them by the conversion factor cited in the original source, if given, or the IPCC conversion factor of 0.47.

For all forest types, plot‐level AGB values were calculated in the original sources by aggregating tree‐level AGB within each plot. Tree‐level AGB was estimated based on diameter at breast height, tree height (if available) and species‐specific wood density. The set of allometric equations (Chave et al., 2005, 2014; Feldpausch et al., 2012; Talbot et al., 2014) used in the original sources were carefully selected to account for climatic factors such as different levels of precipitation and bioclimatic stress.

### 2.2 Calculation of ∆AGB rates per forest type

**Figure 1:**
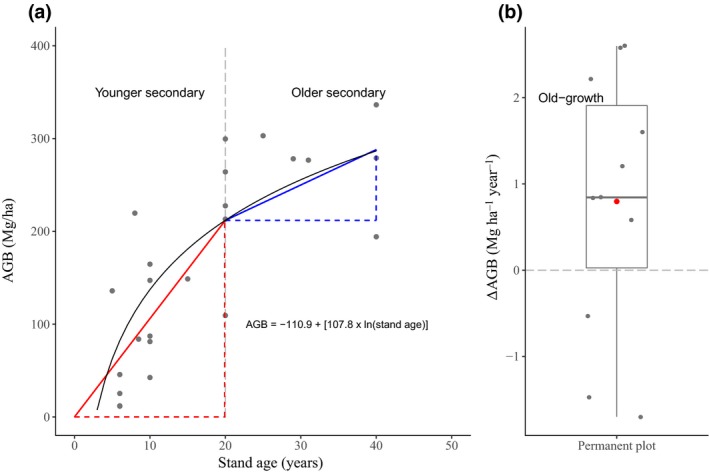
Examples of (a) an aboveground biomass (AGB)–stand age relationship in a given chronosequence in a secondary forest and (b) ∆AGB rates in a given permanent plot in an old‐growth forest. In (a), grey dots indicate AGB plot values. ∆AGB rates are calculated by obtaining two slopes per growth curve (the black curve): one for younger secondary forests (0–20 years; red line) and another one for older secondary forests (20 years to the maximum age available at a given site; blue line). In (b), grey dots indicate ∆AGB rates derived from consecutive census in a given plot, with the red dot showing the mean value across censuses for that plot

∆AGB rates were derived separately for younger secondary forests, older secondary forests, old‐growth forests and managed/logged forests. For younger and older secondary forests, a mixed‐effects modelling framework was applied to model AGB as a function of stand age (fixed effect) and chronosequence sites (random effect). For this, we used the *lme4* package (Bates, Maechler, Bolker, & Walker, 2015) in R v.3.4.0 (R Core Team, 2017). Stand age was ln‐transformed to account for the nonlinear increase in AGB with stand age. Subsequently, plot AGB for each chronosequence was modelled as a function of stand age, including a random intercept and slope (Figure 1a).

For younger and older secondary forests, site‐specific models, that is, models with a site‐specific intercept and slope based on the random effects, were used to derive ∆AGB rates per chronosequence. For younger secondary forests, we did so by predicting AGB at 20 years and then dividing this value by 20 to obtain the ∆AGB rate (Figure 1a; slope of the red line). As such, we assumed a linear increase in AGB over the first 20 years of succession, which has been observed in some secondary tropical forests (Alves et al., 1997; Saldarriaga, West, Tharp, & Uhl, 1988). This assumption is not always accurate (e.g. when some biomass remains following disturbance or when succession is delayed), and our calculated rates, therefore, will not always accurately represent instantaneous ∆AGB rates for stands ≤20 years. However, this approach yields rate estimates that should provide, on average, unbiased average estimates over the first 20 years of forest regrowth when applied in a bookkeeping context. For older secondary forests, AGB was predicted at 20 years (or the youngest age after 20 years) and at the maximum stand age available. Following this, site‐specific ∆AGB rates were calculated by subtracting AGB at 20 years from AGB at the maximum stand age, then dividing it by the difference in stand age (Figure 1a; slope of the blue line).

For old‐growth forests and for managed/logged forests, permanent plots from which ∆AGB rates were obtained were treated as single sites. ∆AGB rates were weighted according to total monitoring period and plot size, following the weighting procedures determined in the original sources (Brienen et al., 2015; Lewis et al., 2009; Qie et al., 2017; Rutishauser et al., 2015), unless all plots in an ecozone presented the same monitoring period and plot size, such as for plots in managed/logged forests in Africa (Gourlet‐Fleury et al., 2013). For permanent plots with two or more census intervals and two or more ∆AGB rates derived from these, we used the mean ∆AGB rate (Figure 1b).

### 2.3 Derivation of IPCC default ∆AGB rates

To derive IPCC ∆AGB default rates, site‐specific ∆AGB rates were averaged per continent, ecozone and forest type (younger secondary, older secondary and old‐growth). Following IPCC requirements, data from managed/logged forest data, when available, were combined with the older secondary forest type. Default ∆AGB rates were calculated for categories (i.e. combinations of continent, ecozone and forest type) with sufficient data only (≥2 chronosequences or permanent plots per category). For younger and older secondary forest categories, we included standard deviations (*SD*) and confidence intervals (CI; 95%) as measures of variation. For old‐growth forest categories, we calculated the weighted *SD* and bootstrapped CI (95%, 1,000 repetitions with replacement).

For categories with insufficient data, we used default rates from the same ecozone and forest type from another continent. If default rates from the other two continents were available, we chose the value that more closely aligned with the default value(s) of a different forest type in the ecozone and continent of interest. If no data were available across all three continents, we recommended using the IPCC 2006 default rates. For the latter cases, we did not differentiate between old‐growth forests and secondary forests >20 years, per the IPCC 2006 default rates. Categories with recommended rates can be found in Appendix S1. For ecozones with sufficient data for secondary forests but insufficient data for old‐growth forests, only default rates for secondary forests were derived (e.g. tropical moist forests in Africa, Table 1).

**Table 1:** Refined default aboveground net biomass change (∆AGB) rates

Ecozone	Continent	Forest type^a^	Aboveground biomass change (∆AGB) (Mg ha^−1^ year^−1^)				No. of chronosequences and/or permanent plots
			Mean ∆AGB	Median ∆AGB	*SD*	CI (95%)	
Tropical rainforest	Africa	YS	7.6	3.5	5.9	4.6, 10.6	15
		OS	3.5	1.9	3.3	1.5, 5.5	10
		OG	1.3	1.7	3.5	0.5, 2.1	77
	North and South America	YS	5.9	5.0	2.5	5.1, 6.7	42
		OS	2.3	2.1	1.1	2.0, 2.6	39
		OG	1.0	0.9	2.0	0.6, 1.4	248
	Asia	YS	3.4	2.1	3.9	0.5, 6.3	7
		OS	2.7	2.7	3.1	−1.6, 7.0	2
		OG	0.7	0.8	2.2	0.1, 1.3	66
Tropical moist forest	Africa	YS	2.9	2.9	1.0	1.5, 4.3	2
		OS	0.9	0.9	0.7	−0.1, 1.9	2
	North and South America	YS	5.2	4.5	2.3	4.2, 6.2	21
		OS	2.7	2.2	1.7	1.9, 3.5	18
		OG	0.4	0.8	2.1	−0.7, 1.5	19
	Asia	YS	2.4	2.4	0.3	2.0, 2.8	2
Tropical dry forest	North and South America	YS	3.9	3.1	2.4	2.0, 5.8	6
		OS	1.6	1.5	1.1	0.6, 2.6	5
Tropical mountain system	Africa	YS	5.5	5.5	6.8	−3.9, 14.9	2
	North and South America	YS	4.4	4.0	1.6	3.1, 5.7	6
		OS	1.8	1.5	0.8	1.0, 2.6	4
		OG	0.5	0.1	1.9	−0.9, 1.9	6
	Asia	YS	2.9	2.9	0.1	2.8, 3.0	5
		OS	1.1	1.2	0.4	0.7, 1.5	5
		OG	−0.7	−0.3	3.1	−3.2, 1.8	5
Subtropical humid forest	Asia	YS	2.5	2.2	0.8	1.7, 3.3	4
		OS	1.0	0.7	0.9	0.4, 1.6	8
Subtropical mountain system	Asia	YS	2.5	2.5	0.03	2.5, 2.5	2
		OS	0.5	0.4	0.3	0.3, 0.7	12

Note

Forest types include younger secondary forests (YS), older secondary forests (OS) and old‐growth forests (OG). Refined IPCC default ∆AGB rates consist of mean ∆AGB and *SD* (in grey). See Appendix S1 for a complete version of the table that includes recommended rates for categories without data.

a IPCC‐defined forest type categories are ‘Secondary ≤20 years’ (younger secondary forests), “Secondary >20 years” (older secondary forests) and “Primary” (old‐growth forests).

## 3 RESULTS

### 3.1 Data availability

Overall, we assembled a database of 176 chronosequences (consisting of 1,924 plots) of secondary forests and 536 permanent plots (1,324 census intervals) of old‐growth or managed/logged forests. Within chronosequences, younger secondary forests were better represented than older secondary forests (65.7% and 34.3% of plots respectively). Of all permanent plots, the majority were located in old‐growth forests (79.1%, 1,212 census intervals in total), as opposed to managed/logged forests (20.9%, 112 census intervals in total).

**Figure 2:**
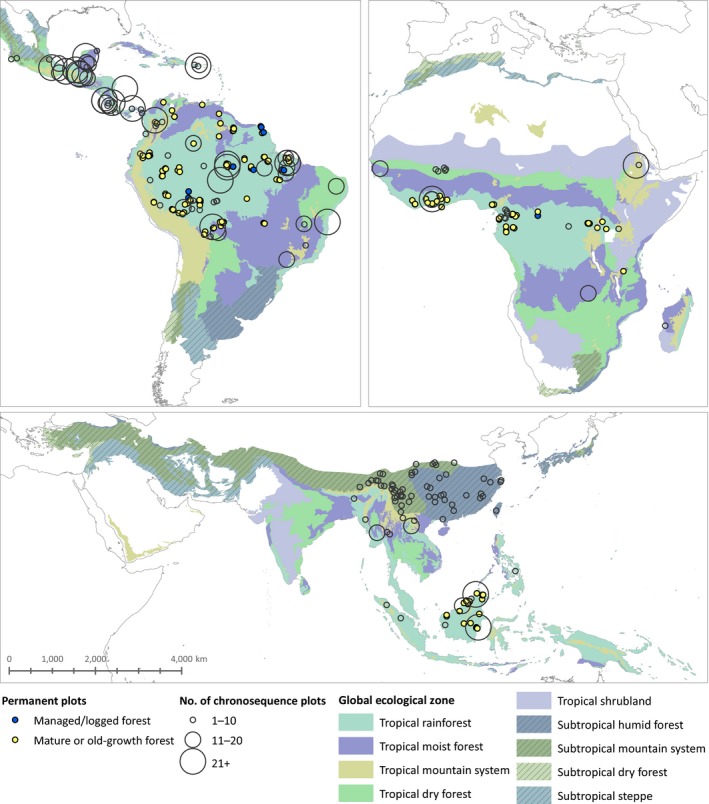
Distribution of chronosequences and permanent plots. Coloured areas show the extent of global ecological zones (according to FAO, 2012) included in this study; subtropical ecozones are hatched. Chronosequences are indicated with hollow circles; symbol size varies with the number of plots per chronosequence. Permanent plots are indicated with blue (managed/logged forests) and yellow (old‐growth forests) circles

For secondary forests, 43.8% of the chronosequences were situated in North and South America (excluding continental United States), 15.3% in Africa and 40.9% in Asia. For permanent plots in old‐growth forests, 64.6% of plots were situated in North and South America, 18.6% in Africa and 16.7% in Asia. For permanent plots in managed/logged forests, 75% of plots were situated in North and South America and 25% in Africa. Overall 67.6% of chronosequences and 100% of permanent plots were situated in tropical, as opposed to subtropical ecozones (Figure 2).

In North and South America, of 77 chronosequences, 27.3% had >20 plots, 19.5% had between 11 and 20 plots and 53.2% had ≤10 plots. In Africa, of 27 chronosequences, 7.4% had >20 plots, 22.2% had between 11 and 20 plots and 70.4% had ≤10 plots. In Asia, of 72 chronosequences, 2 had >20 measurements each, 3 had between 11 and 20 measurements and the remaining majority (93.1%) had ≤10 plots.

### 3.2 Default ∆AGB rates per IPCC forest type

We derived new default ∆AGB rates for natural forests per continent, ecozone and forest type (Table 1). Across all continents and ecozones, our default ∆AGB rates for younger secondary forests were higher than for older secondary forests, which in turn were higher than rates for old‐growth forests. In tropical rainforests, default rates for all forest types were higher in Africa than in North and South America and Asia. In tropical mountain systems, default rates for younger secondary forests were also higher in Africa than in the other continents (Table 1). Default ∆AGB rates in old‐growth forests ranged from −0.7 (−0.1, 1.9) in tropical mountain systems in Asia to 1.3 (0.5, 2.1) Mg ha^−1^ year^−1^ in tropical rainforests in Africa. In individual census intervals, negative rates were reported for all ecozones and continents, but default rates for old‐growth forests tended to be significantly positive and especially so where sufficient sample size was available to assess change with a high degree of confidence (Table 1).

### 3.3 Comparison with IPCC 2006 default rates in selected ecozones

We compared our refined rate estimates to previous IPCC 2006 default ∆AGB rates for three ecozones (tropical rainforests, tropical moist forests and tropical mountain systems) with the highest data availability, and for which default rates were derived across all continents for at least one forest type.

For younger secondary forests, our refined rate estimates were lower than the IPCC 2006 default rates for forests <20 years old, with the exception of tropical mountain systems in North and South America (1 Mg ha^−1^ year^−1^ higher) and Africa (2 Mg ha^−1^ year^−1^ higher).

**Figure 3:**
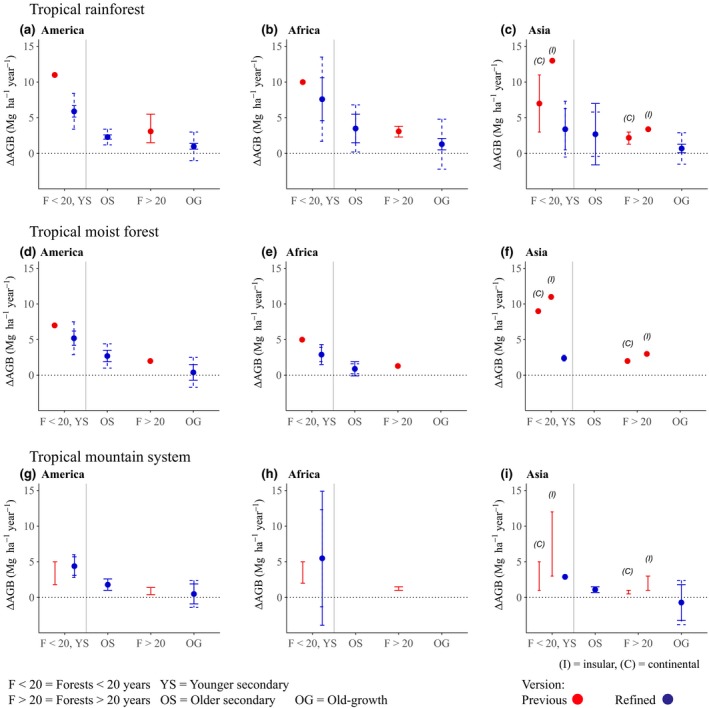
Comparison of previous IPCC 2006 default aboveground net biomass change (∆AGB) rates with refined default rates per continent and forest type in tropical rainforests (a, b, c), tropical moist forests (d, e, f) and tropical mountain systems (g, h, i). Previous (in red) ∆AGB rates (Mg ha^−1^ year^−1^) were divided into forests ≤20 years and forests >20 years old. Our refined (in blue) ∆AGB rates are divided into younger secondary forests, older secondary forests and old‐growth forests. Vertical continuous lines represent ranges for previous default rates and CI (95%) for refined default rates, and vertical dashed lines represent *SD*. For Asia, previous rates were divided into continental and insular values. The grey vertical line divides forests ≤20 years and younger secondary forests from the other forest types

Our refined rates for the new forest types (older secondary forests and old‐growth forests) that replaced forests >20 years old partially aligned with IPCC 2006 default rates (Figure 3). In all cases, our rates for old‐growth forests were more conservative (i.e. smaller net positive gains) than the IPCC 2006 default rates for all forests >20 years old. For older secondary forests, our rates in North and South America and in Africa were higher than the IPCC 2006 default rates for forests >20 years old, with the exception of tropical rainforests in North and South America (0.8 Mg ha^−1^ year^−1^ lower; Figure 3a) and tropical moist forests in Africa (0.4 Mg ha^−1^ year^−1^ lower; Figure 3e). In Asia, our rates for older secondary forests were lower than for the IPCC 2006 default rates previously calculated separately for insular and continental areas (Figure 3c,f,i). The distinction between insular and continental rates for Asia is residual from the IPCC 2006 rates and was not continued in our estimates, due to limited data availability. Across all forest types, standard deviations tended to be higher for rates obtained from forest categories with fewer sites. For example, in tropical rainforests, the *SD* for younger secondary forests in Africa (15 sites) was more than double the *SD* for the same forest category in North and South America (42 sites).

### 3.4 ∆AGB rates per forest type for selected ecozones

**Figure 4:**
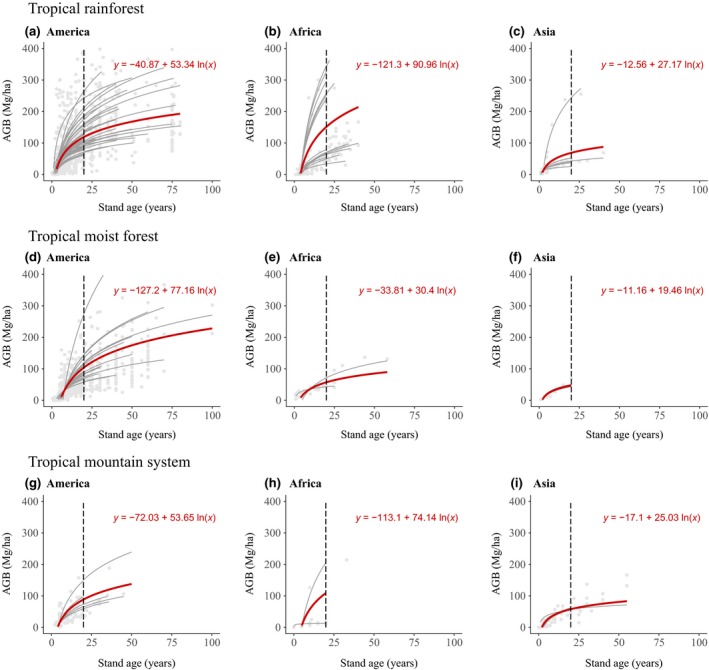
Relationships between aboveground biomass (AGB) and stand age in tropical rainforests (a, b, c), tropical moist forests (d, e, f) and tropical mountain systems (g, h, i) for secondary forests. AGB plots and chronosequences are represented in grey dots and grey curves respectively. Overall relationships between AGB and stand age for each ecozone are presented in red. The dashed vertical line divides the graph into younger secondary (≤20 year) and older secondary (>20 years) forests. Data from managed/logged forests were not included in this figure. For other ecozones, data were not available across all three continents

In secondary forests, AGB–stand age relationships varied strongly between continents and ecological zones (Figure 4). A complete list of AGB–stand age relationships for secondary forests can be found in Appendix S2. Across all continents, tropical rainforests (Figure 4a,b,c) showed the highest ∆AGB rates in comparison with other ecozones. In North and South America, where data availability was highest, AGB at 20 years varied from 88.7 Mg/ha (tropical mountain system) to 118.9 Mg/ha (tropical rainforest). Variation was stronger in Africa, where AGB after 20 years ranged from 57.3 Mg/ha (tropical moist forest) to 151.2 Mg/ha (tropical rainforest). Asia showed lower variation within ecological zones, with AGB after 20 years ranging from 47.1 Mg/ha (tropical moist forest) to 68.8 Mg/ha (tropical rainforest).

**Figure 5:**
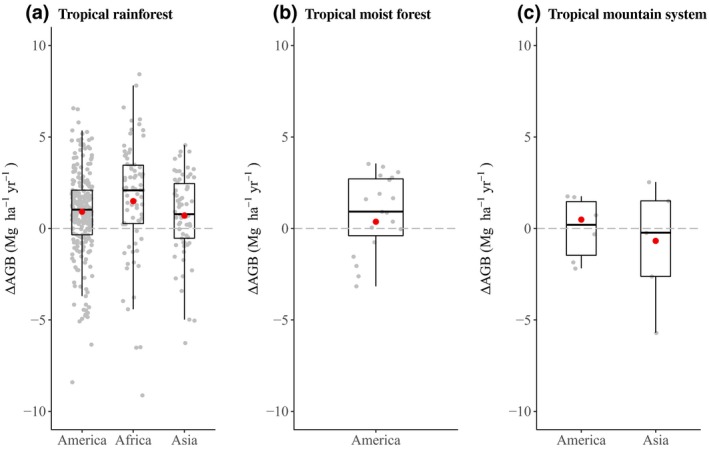
Aboveground net biomass change (∆AGB) rates in old‐growth (a) tropical rainforests, (b) tropical moist forests and (c) tropical mountain systems. Plot‐specific ∆AGB rates are represented in grey. Red dots represent the mean ∆AGB rate per ecozone. Two values (−16.24 and −10.84) in tropical rainforests in Africa were excluded from (a)

For old‐growth forests, mean ∆AGB rates were positive with the exception of tropical mountain systems in Asia (Figure 5c). Site‐specific negative ∆AGB rates were present across all three ecozones. In such cases, negative rates indicate a period in which biomass loss by mortality has exceeded biomass accumulation by growth and recruitment over a period of time. Mean rates in old‐growth tropical rainforests were highest in Africa, followed by North and South America, then Asia (Table 1). Mean rates in tropical mountain systems ranged from −0.7 Mg ha^−1^ year^−1^ in Asia to 0.5 Mg ha^−1^ year^−1^ in North and South America. In North and South America, old‐growth tropical moist forests showed the lowest rate (0.4 Mg ha^−1^ year^−1^) in comparison with the rates obtained for old‐growth forests in the other two ecozones (Table 1).

**Figure 6:**
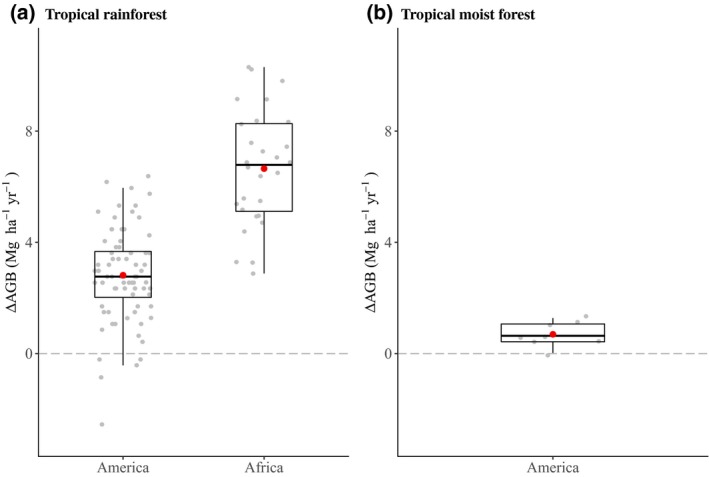
Aboveground net biomass change (∆AGB) rates in managed/logged (a) tropical rainforests and (b) tropical moist forests. Plot‐specific ∆AGB rates are represented in grey. Red dots represent the mean ∆AGB rate per ecozone

For managed/logged forests, ∆AGB rates were available only for tropical rainforests in North and South America and in Africa and for tropical moist forests in North and South America. For tropical rainforests (Figure 6a), the mean rate in Africa was more than twice as high as the mean rate in North and South America. In America, managed/logged forests in tropical rainforests had a higher mean rate than tropical moist forests (2.8 and 0.7 Mg ha^−1^ year^−1^ respectively).

## 4 DISCUSSION

### 4.1 Refined IPCC default ∆AGB rates across forest types

Our refined rates were on average 30% smaller than the IPCC 2006 default rates, indicating that the use of the latter may overestimate forest C sequestration. Our rates for younger secondary forests were 30.1% smaller than the IPCC 2006 rates for forests <20 years old. Our rates for older secondary forests were on average neither smaller nor larger than the IPCC 2006 rates for forests >20 years old. Rates for old‐growth forests, however, where on average 79.4% smaller than the IPCC 2006 rates for forests >20 years old. Thus, disaggregating older secondary forests and old‐growth forests from the previous category of forests >100 years has provided us with more nuanced default rates.

Standard deviations per continent and ecozone ranged from 0.03 to 6.8 Mg ha^−1^ year^−1^ in younger secondary forests, from 0.3 to 3.3 in older secondary forests and from 1.9 to 3.5 in old‐growth forests. The large variability in SDs is partly due to the limited amount of sites or plots in many categories, which can result in a low *SD* if all chronosequences or plots are under similar site conditions. On the other hand, high SDs are not an unexpected result from combining plot measurements from forests that differ in their composition and site‐specific factors. This variability can be observed in tropical mountain systems, for which more chronosequences and permanent plots are needed. Due to the variability in forests within ecozones, SDs and confidence intervals in categories with a limited number of sites should be used with caution, as these values would likely change with the addition of more sites.

Our refined rates can be used for entire ecozones per forest type, therefore, they are suitable for Tier 1 calculations. These rates should only be used in the absence of country‐specific emission/removal factors (Tier 2) and/or local and detailed ∆AGB data (Tier 3; IPCC, 2006). Tropical countries reporting at Tier 1 level, but with substantial or highly uncertain estimates of AGB and ∆AGB in their natural forests, should strive to collect country‐level data to report at higher tier levels.

Our methodology can be further refined for Tier 2 and Tier 3 calculations by accounting for deviations resulting from within‐ecozone variation due to site conditions such as climate (e.g. precipitation, temperature), soil fertility, species composition, the presence of remnant trees and previous land use, all of which influence ∆AGB (Chazdon, 2014; Feldpausch, Rondon, Fernandes, Riha, & Wandelli, 2004; N'Guessan et al., 2019; Poorter et al., 2016; Rozendaal et al., 2017). Similarly, given the variability in ∆AGB across forest succession, forest types could be further disaggregated into smaller age classes, in particular among older secondary forests.

While our study focuses only on the C pool of living biomass and its change in natural forests, countries with a substantial amount of planted forests should also consider them when describing this pool at Tier 1 level. Default values and methods for planted forests are included in the 2006 IPCC Guidelines (IPCC, 2006), and have also been updated in the 2019 Refinement (IPCC, 2019). Furthermore, other C pools, such as dead organic matter or soil organic matter, should also be accounted for when estimating total forest C pools and sinks. Methods for estimating these pools are included in the 2006 IPCC Guidelines, and have also been partly updated in the 2019 Refinement.

### 4.2 Methodological implications

#### 4.2.1 Secondary forests—use of chronosequences

For secondary forests, we derived ∆AGB rates from chronosequences, an approach that is typically applied to estimate AGB accumulation during secondary forest succession (e.g. Feldpausch, Conceicao Prates‐Clark, Fernandes, & Riha, 2007; Poorter et al., 2016). However, this approach has limitations. By substituting space for time, we assume that all measurements have been affected in the same way by biotic and abiotic conditions (Johnson & Miyanishi, 2008), which may not be the case. To obtain actual ∆AGB rates in secondary forests in future refinements, long‐term monitoring through repeated measurements of secondary forest plots is needed. While this has been carried out in some sites (e.g. Chazdon et al., 2007; Feldpausch et al., 2007; Rozendaal et al., 2017), such data were not available for many sites thus far, and data that were available deviated from chronosequence predictions in some sites (Feldpausch et al., 2007).

The compiled chronosequences consisted mostly of plots in stands below 20 years of age; thus, estimates for older secondary forests rely on less data. Furthermore, of all plots in older secondary forests, only 19.4% had stand ages over 60 years. Because of these limitations in data availability, we decided to not extend the modelled AGB–stand age relationships beyond the maximum age available per ecozone (Appendix S2) instead of extending these relationships until the cut‐off at 100 years. The rates obtained for older secondary forests will have an upward bias; as more data in older secondary forests become available, in particular in stands over 60 years old, ∆AGB estimates in older secondary forests should be further refined.

As expected, ∆AGB estimates in young secondary forests were higher compared to old‐growth forests. There is high C sequestration potential in secondary forests through reforestation and forest restoration (Chazdon et al., 2016); however, due to their vulnerability and rapid turnover, as well as a lack of mechanisms for their conservation (Vieira et al., 2014), secondary forests remain vulnerable to deforestation and degradation.

#### 4.2.2 Old‐growth and Managed/logged forests—use of permanent plots

In old‐growth forests, site‐specific ∆AGB rates spanned from positive to negative values. Site‐specific positive rates may occur in stands recovering from past disturbance and/or in response to global change processes such as changes in atmospheric CO_2_ concentration or N deposition (Lewis, Malhi, & Phillips, 2004; Luo, 2007). Site‐specific negative rates may account for particular periods when biomass loss was higher than biomass gain due to stochastic processes such as tree mortality resulting from natural gap phase dynamics, or due to exceptional and/or repeated droughts and climate variability (Brienen et al., 2015; Feldpausch et al., 2016; Phillips et al., 2009; Qie et al., 2017). The plot‐to‐plot variability makes it clear that large sample sizes are needed in order to better constrain old‐growth ecosystem biomass trajectories. While our results indicate that old‐growth forests are on average small C sinks per unit area, they become a significant component of the planetary carbon balance due to their large extent and for large amounts of biomass they store (Pan et al., 2011).

In managed/logged forests, an overwhelming majority of sites (95.5%) had positive site‐specific ∆AGB rates. Similar to old‐growth forests, the few sites in managed/logged forest with negative rates are associated with stochastic events such as tree mortality (Rutishauser et al., 2015). High positive site‐specific rates are expected from managed/logged sites, as they are obtained from remaining stands after logging. These rates do not account for released C by logging or silvicultural practices, which can vary depending on the type of logging techniques (Putz et al., 2008).

Given the growing extent of tropical forests with constant anthropogenic disturbances (Lewis, Edwards, & Galbraith, 2015), further research should be done on ∆AGB in managed/logged forests, particularly in relation to the effects of different types of logging practices. Once more data on this forest type become available, it will be possible, and advisable, to disaggregate estimates for managed/logged forests from older secondary forests.

Furthermore, more plots in degraded forests are necessary to understand how degradation affects ∆AGB. Currently, our estimates do not account for level of degradation. There are studies that focus on the effects of forest degradation on AGB (Berenguer et al., 2014; Chaplin‐Kramer et al., 2015); but effects on ∆AGB remain largely unknown and should be further explored. For this reason, countries with a large extent of degraded forests should consider our estimates as a first step, and account for the effect that degraded forests may have on ∆AGB through the establishment and monitoring of plots in degraded forests.

### 4.3 Improving ∆AGB data availability

**Figure 7:**
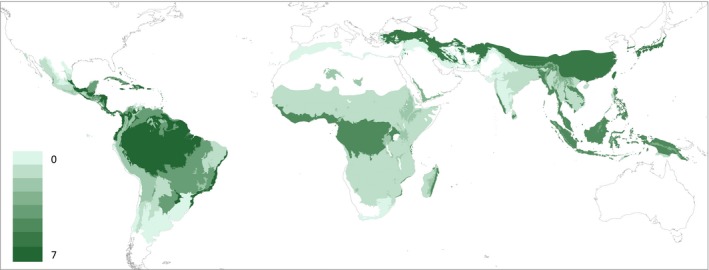
Chronosequence and permanent plot density per 100,000 km^2^ of natural forests in tropical and subtropical ecozones. Extent of natural forests were obtained from Schulze, Malek, and Verburg (2019) and combined with FAO (2012) to obtain coarse estimates of natural forest area per ecozone. A full list of chronosequence and plot density can be found in Appendix S3

Data availability varied across ecozones and continents (Figure 7). More data were available in tropical ecozones than in subtropical ecozones, and the latter had better data availability in Asia in comparison to the other continents. There were no chronosequences nor permanent plots available in subtropical dry forests or subtropical steppes in any of the continents. In addition, there were no data available for tropical shrublands and subtropical humid forests in North and South America, subtropical humid forests and subtropical mountain systems in Africa and tropical shrublands in Asia.

To derive large‐scale estimates, a high number of chronosequences and permanent plots per ecozone is recommended to ensure representative estimates (Muller‐Landau et al., 2014; Phillips, Lewis, Higuchi, & Baker, 2016; Poorter et al., 2016). Even though tropical rainforests had higher chronosequence and permanent plot densities across all continents, these densities are still relatively low (6.2, 4.7 and 4.3 chronosequences and permanent plots per 100,000 km^2^ of natural forests in North and South America, Africa and Asia respectively). Given the extent of natural forests in tropical forest ecozones, their high inherent C sequestration potential (particularly in secondary forests) and their vulnerability to global change, more carefully positioned plots are needed to enhance the long‐term monitoring of these forests at different successional stages. On the other hand, natural forests in ecozones with lower density of chronosequences and permanent plots should also be prioritized in future research (Figure 7; Appendix S3). For example, little is known about ∆AGB in low‐biomass forests in tropical shrublands, even though this ecozone accounts for a substantial land area in Africa (approximately 5.95 × 10^12^ km^2^).

The various threats to tropical forests posed by global change processes themselves means that it would be naïve to simply assume that past records are likely to be a good guide to future behaviour of these forests (e.g. Cavaleri, Reed, Smith, & Wood, 2015): the future C balance of tropical and subtropical forests under a changing climate remains unknown. There is, however, already some evidence that these sinks are threatened by global change pressures and have been declining recently in some regions (Brienen et al., 2015; Qie et al., 2017). Expanded and careful long‐term monitoring with permanent plots will be needed to understand the changing carbon dynamics of the world's tropical and subtropical forests.

### 4.4 Future possibilities for improvement

To make use of more field data, AGB plots without stand age could be used in conjunction with a reliable stand age map to derive ∆AGB estimates. There have been advances in the elaboration of stand age maps (e.g. Poulter et al., 2018); however, such maps are currently not available in the resolution nor certainty required. Furthermore, disaggregating ∆AGB as a result of natural forest dynamics from forest degradation remains a challenge (Bustamante et al., 2016; Mitchell, Rosenqvist, & Mora, 2017).

For categories for which rates could not be derived due to insufficient data, there is promise in using remote sensing (RS) data to monitor ∆AGB at a large scale. This could be achieved through consistent monitoring of forest cover change and biomass change at high spatial and temporal resolutions. Current global or pantropical RS products provide valuable information regarding forest cover gain or biomass change, but do so at medium‐to‐low spatial resolutions (e.g. Song et al., 2018) and for one particular time period instead of annually (e.g. Hansen et al., 2013). For example, the aboveground C density change map of Baccini et al. (2017) accounts for net change from 2003 until 2014, and, due to its methodology and low spatial resolution, does not distinguish between C density change from natural forest dynamics or from anthropogenic processes such as deforestation and degradation.

Evolving initiatives on AGB estimation such as the Global Ecosystem Dynamics Investigation mission (Dubayah et al., 2014), which aims to provide periodic AGB density estimates at a global scale, will facilitate our access to spatially explicit and multitemporal AGB estimates. In addition, interdisciplinary approaches that integrate AGB and ∆AGB plot data with RS data from the start will prove to be useful for future updates.

## 5 CONCLUSIONS

As part of the 2019 Refinement to the 2006 IPCC Guidelines for National Greenhouse Gas Inventories (IPCC, 2019), we provide a rigorous refinement of the Tier 1 IPCC 2006 default ∆AGB rates for tropical and subtropical forests by incorporating forest plot data that have become available since the publication of the IPCC 2006 default rates. Our refined rates disaggregate forests >20 years old into older secondary forests and old‐growth forests, and provide measures of variation to account for their uncertainty. These new rates can be used for large‐scale C accounting by governmental bodies, nongovernmental organizations and in scientific research. Due to their spatial coarseness, these rates are not recommended for project‐level monitoring. We present a clear, simple and reproducible approach to derive these rates, and have identified the ecozones for which more research is needed; therefore, these rates can be further refined as more data become available. In this respect, this study should be considered as an important step forward towards quantifying the role of tropical and subtropical forests as C sinks at large scales with higher accuracy.
