# Supplementary material for: Estimating aboveground net biomass change for tropical and subtropical forests: Refinement of IPCC default rates using forest plot data
Source: Glob Chang Biol. 2019 Aug 16;25(11):3609–24. doi: 10.1111/gcb.14767 (PMC6852081; doi:10.1111/gcb.14767)
Supplement: Supplementary file 1 [file GCB-25-3609-s001.pdf]

## Supporting information

**Appendix 1.** Default rates of aboveground net biomass change (ΔAGB; in Mg $ha^{-1}$ $yr^{-1}$). Forest types consist of old-growth (OG), older secondary forests (OS) and younger secondary forests (YS).

| **Ecological Zone** | **Continent** | **Forest Type** [a] | **ΔAGB (Mg $ha^{-1}$ $yr^{-1}$)** | | **Source of recommendation** | **References** |
|---|---|---|---|---|---|---|
| | | | **Mean** | **SD** | | |
| Tropical rainforest | Africa | OG | 1.3 | 3.5 | | [1, 2] |
| | | OS | 3.5 | 3.3 | | [3-8] |
| | | YS | 7.6 | 5.9 | | [3-7, 9] |
| | North and South America | OG | 1 | 2 | | [2, 10, 11] |
| | | OS | 2.3 | 1.1 | | [3, 4, 12-15] |
| | | YS | 5.9 | 2.5 | | [3, 4, 6, 12-14] |
| | Asia | OG | 0.7 | 2.2 | | [2, 16] |
| | | OS | 2.7 | 3.1 | | [3, 4, 17] |
| | | YS | 3.4 | 3.9 | | [3, 4, 17-19] |
| Tropical moist forest | Africa | OG | 0.4 | NA | Recommendation from a different region | |
| | | OS | 0.9 | 0.7 | | [20, 21] |
| | | YS | 2.9 | 1.0 | | [20, 21] |
| | North and South America | OG | 0.4 | 2.1 | | [2, 10, 11] |
| | | OS | 2.7 | 1.7 | | [3, 4, 12, 13, 15, 22] |
| | | YS | 5.2 | 2.3 | | [3, 4, 12, 13, 22] |
| | Asia | OG | 0.4 | NA | Recommendation from a different region | |
| | | OS | 0.9 | NA | Recommendation from a different region | |
| | | YS | 2.4 | 0.3 | | [3, 4] |
| Tropical dry forest | Africa | OG | - | - | | |
| | | OS | 1.6 | NA | Recommendation from a different region | |
| | | YS | 3.9 | NA | Recommendation from a different region | |
| | North and South America | OG | - | - | | |
| | | OS | 1.6 | 1.1 | | [12, 13] |
| | | YS | 3.9 | 2.4 | | [12, 13, 23] |
| | Asia | OG | - | - | | |
| | | OS | 1.6 | NA | Recommendation from a different region | |
| | | YS | 3.9 | NA | Recommendation from a different region | |
| Tropical shrublands | Africa | OG * | 0.9 (0.2-1.6) | NA | Recommendation from IPCC 2006 rates | [24] |
| | | OS * | 0.9 (0.2-1.6) | NA | Recommendation from IPCC 2006 rates | [24] |
| | | YS | 0.2-0.7 | NA | Recommendation from IPCC 2006 rates | [24] |
| | North and South America | OG * | 1.0 | NA | Recommendation from IPCC 2006 rates | [24] |
| | | OS * | 1.0 | NA | Recommendation from IPCC 2006 rates | [24] |
| | | YS | 4.0 | NA | Recommendation from IPCC 2006 rates | [24] |
| | Asia (continental) | OG * | 1.3 (1.0-2.2) | NA | Recommendation from IPCC 2006 rates | [24] |
| | | OS * | 1.3 (1.0-2.2) | NA | Recommendation from IPCC 2006 rates | [24] |
| | | YS | 5.0 | NA | Recommendation from IPCC 2006 rates | [24] |
| | Asia (insular) | OG * | 1.0 | NA | Recommendation from IPCC 2006 rates | [24] |
| | | OS * | 1.0 | NA | Recommendation from IPCC 2006 rates | [24] |
| | | YS | 2.0 | NA | Recommendation from IPCC 2006 rates | [24] |
| Tropical mountain system | Africa | OG | 0.5 | NA | Recommendation from a different region | |
| | | OS | 1.8 | NA | Recommendation from a different region | |
| | | YS | 5.5 | 6.8 | | [25-27] |
| | North and South America | OG | 0.5 | 1.9 | | [2, 10, 11] |
| | | OS | 1.8 | 0.8 | | [3, 4, 12, 13] |
| | | YS | 4.4 | 1.6 | | [3, 4, 12, 13, 22] |
| | Asia | OG | -0.7 | 3.1 | | [2, 16] |
| | | OS | 1.1 | 0.4 | | [3, 4, 28, 29] |
| | | YS | 2.9 | 0.1 | | [3, 4, 28-30] |

| | | | | | | |
|---|---|---|---|---|---|---|
| Subtropical humid forest | Africa | OG | - | - | | |
| | | OS | 1.0 | NA | Recommendation from a different region | |
| | | YS | 2.5 | NA | Recommendation from a different region | |
| | North and South America | OG | - | - | | |
| | | OS | 1.0 | NA | Recommendation from a different region | |
| | | YS | 2.5 | NA | Recommendation from a different region | |
| | Asia | OG | - | - | | |
| | | OS | 1.0 | 0.9 | | [3, 4, 31] |
| | | YS | 2.5 | 0.8 | | [3, 4, 31] |
| Subtropical dry forest | Africa | OG * | 1.8 (0.6-3.0) | NA | Recommendation from IPCC 2006 rates | [24] |
| | | OS * | 1.8 (0.6-3.0) | NA | Recommendation from IPCC 2006 rates | [24] |
| | | YS | 2.4 (2.3-2.5) | NA | Recommendation from IPCC 2006 rates | [24] |
| | North and South America | OG * | 1.0 | NA | Recommendation from IPCC 2006 rates | [24] |
| | | OS * | 1.0 | NA | Recommendation from IPCC 2006 rates | [24] |
| | | YS | 4.0 | NA | Recommendation from IPCC 2006 rates | [24] |
| | Asia (continental) | OG * | 1.5 | NA | Recommendation from IPCC 2006 rates | [24] |
| | | OS * | 1.5 | NA | Recommendation from IPCC 2006 rates | [24] |
| | | YS | 6.0 | NA | Recommendation from IPCC 2006 rates | [24] |
| | Asia (insular) | OG * | 2.0 | NA | Recommendation from IPCC 2006 rates | [24] |
| | | OS * | 2.0 | NA | Recommendation from IPCC 2006 rates | [24] |
| | | YS | 7.0 | NA | Recommendation from IPCC 2006 rates | [24] |
| Subtropical steppe | Africa | OG * | 0.9 (0.2-1.6) | NA | Recommendation from IPCC 2006 rates | [24] |
| | | OS * | 0.9 (0.2-1.6) | NA | Recommendation from IPCC 2006 rates | [24] |
| | | YS | 1.2 (0.8-1.5) | NA | Recommendation from IPCC 2006 rates | [24] |
| | North and South America | OG * | 1.0 | NA | Recommendation from IPCC 2006 rates | [24] |
| | | OS * | 1.0 | NA | Recommendation from IPCC 2006 rates | [24] |
| | | YS | 4.0 | NA | Recommendation from IPCC 2006 rates | [24] |
| | Asia (continental) | OG * | 1.3 (1.0-2.2) | NA | Recommendation from IPCC 2006 rates | [24] |
| | | OS * | 1.3 (1.0-2.2) | NA | Recommendation from IPCC 2006 rates | [24] |
| | | YS | 5.0 | NA | Recommendation from IPCC 2006 rates | [24] |
| | Asia (insular) | OG * | 1.0 | NA | Recommendation from IPCC 2006 rates | [24] |
| | | OS * | 1.0 | NA | Recommendation from IPCC 2006 rates | [24] |
| | | YS | 2.0 | NA | Recommendation from IPCC 2006 rates | [24] |
| Subtropical mountain system | Africa | OG | - | - | | |
| | | OS | 0.5 | NA | Recommendation from a different region | |
| | | YS | 2.5 | NA | Recommendation from a different region | |
| | North and South America | OG | - | - | | |
| | | OS | 0.5 | NA | Recommendation from a different region | |
| | | YS | 2.5 | NA | Recommendation from a different region | |
| | Asia | OG | - | - | | |
| | | OS | 0.5 | 0.3 | | [3, 4, 32] |
| | | YS | 2.5 | 0.03 | | [3, 4, 32] |

*Recommendation based on IPCC 2006 rates for forests > 20 years

[a] IPCC-defined forest type categories are “Primary” (old-growth forests), “Secondary >20 years” (older secondary forests) and “Secondary ≤20years” (younger secondary forests)

References
1. S. L. Lewis et al. (2009) Increasing carbon storage in intact African tropical forests. Nature 457, 1003-1006.
2. G. Lopez-Gonzalez, S. L. Lewis, M. Burkitt, O. L. Phillips (2011) ForestPlots.net: a web application and research tool to manage and analyse tropical forest plot data. Journal of Vegetation Science 22, 610-613.
3. K. J. Anderson-Teixeira et al. (2018) forc-db/ForC: Version for IPCC 2019 updated default tables for biomass growth of natural forests in the (sub)tropics.
4. K. J. Anderson-Teixeira et al. (2018) ForC: a global database of forest carbon stocks and fluxes. Ecology 99, 1507.
5. P. A. Omeja, J. S. Lwanga, J. Obua, C. Chapman (2011) Fire control as a simple means of promoting tropical forest restoration. Tropical Conservation Science 4, 287-299.
6. C. A. Palm et al., "Carbon sequestration and trace gas emissions in slash-and-burn and alternative land uses in the humid tropics," (Nairobi, Kenya, 1999).
7. A. E. N'Guessan et al. (2019) Drivers of biomass recovery in a secondary forested landscape of West Africa. Forest Ecology and Management 433, 325-331.
8. S. Gourlet-Fleury et al. (2013) Tropical forest recovery from logging: a 24 year silvicultural experiment from Central Africa. Philos Trans R Soc Lond B Biol Sci 368, 20120302.
9. P. S. Thenkabail, E. A. Enclona, M. S. Ashton, C. Legg, M. J. De Dieu (2004) Hyperion, IKONOS, ALI, and ETM+ sensors in the study of African rainforests. Remote Sensing of Environment 90, 23-43.
10. R. J. W. Brienen et al. (2014) Plot Data from: "Long-term decline of the Amazon carbon sink". ForestPlots.NET.
11. R. J. W. Brienen et al. (2015) Long-term decline of the Amazon carbon sink. Nature 519, 344.
12. L. Poorter et al. (2016) Data from: Biomass resilience of Neotropical secondary forests.
13. L. Poorter et al. (2016) Biomass resilience of Neotropical secondary forests. Nature 530, 211-214.
14. C. I. Salimon, I. F. Brown (2000) Secondary forests in Western Amazonia: Significant sinks for carbon released from deforestation? Interciencia 25, 198-202.
15. E. Rutishauser et al. (2015) Rapid tree carbon stock recovery in managed Amazonian forests. Current Biology 25, R787-R788.
16. L. Qie et al. (2017) Long-term carbon sink in Borneo's forests halted by drought and vulnerable to edge effects. Nature Communications 8, 1966-1966.
17. S. A. Mukul, J. Herbohn, F. Firn (2016) Tropical secondary forests regenerating after shifting cultivation in the Philippines uplands are important carbon sinks. Scientific Reports 6, 22483-22483.
18. M. Hiratsuka, T. Toma, R. Diana, D. Hadriyanto, Y. Morikawa (2006) Biomass Recovery of Naturally Regenerated Vegetation after the 1988 Forest Fire in East Kalimantan, Indonesia. JARQ 40, 277-282.
19. J. J. Ewel, P. Chai, L. M. Tsai (1983) Biomass and floristics of three young second-growth forests in Sarawak. The Malaysian Forester 46, 347-364.
20. F. K. Kalaba, C. H. Quinn, A. J. Dougill, R. Vinya (2013) Floristic composition, species diversity and carbon storage in charcoal and agriculture fallows and management implications in Miombo woodlands of Zambia. Forest Ecology and Management 304, 99-109.
21. R. Manlay et al. (2002) Carbon, nitrogen and phosphorus allocation in agro-ecosystems of a West African savanna I. The plant component under semi-permanent cultivation. Agriculture, Ecosystems and Environment 88, 215-232.
22. M. A. Peña, A. Duque (2013) Patterns of stocks of aboveground tree biomass, dynamics, and their determinants in secondary Andean forests. Forest Ecology and Management2 302, 54-61.
23. M. A. Salinas-Mendoza, M. Skutsch, J. C. Lovett, A. Borrego (2017) Carbon emissions from dryland shifting cultivation: a case study of Mexican tropical dry forest. Silva Fennica 51, 1553-1553.
24. IPCC (2006) Volume 4: Agriculture, Forestry and Other Land Use. 2006 IPCC Guidelines for National Greenhouse Gas Inventories.
25. J. Otuoma et al. (2016) Determinants of aboveground carbon offset additionality in plantation forests in a moist tropical forest in western Kenya. Forest Ecology and Management 365, 61-68.
26. K. Giday, G. Eshete, P. Barklund, W. Aertsen, B. Muys (2013) Wood biomass functions for Acacia abyssinica trees and shrubs and implications for provision of ecosystem services in a community managed exclosure in Tigray, Ethiopia. Journal of Arid Environments 94, 80-86.
27. W. Mekurja, E. Veldkamp, M. D. Corre (2010) Restoration of Ecosystem Carbon Stocks Following Exclosure Establishment in Communal Grazing Lands in Tigray, Ethiopia. SSSAJ 75, 246-256.
28. J. W. Tang et al. (1998) A preliminary study on the biomass of secondary tropical forest in Xishuangbanna. Acta Phytoecol. Sin. 22, 489-498.
29. S. Fujiki, S. Nishio, K. Okada, J. Nais, K. Kitayama (2017) Plant communities and ecosystem processes in a succession-altitude matrix after shifting cultivation in the tropical montane forest zone of northern Borneo. Journal of Tropical Ecology 33, 33-49.
30. N. Chan, S. Takeda (2016) The Transition Away From Swidden Agriculture and Trends in Biomass Accumulation in Fallow Forests: Case Studies in the Southern Chin Hills of Myanmar. Mountain Research and Development 36, 320-331.
31. J. Schomakers et al. (2017) Soil and biomass carbon re-accumulation after landslide disturbances. Geomorphology 288, 164-174.
32. C. L. Dang, Z. L. Wu (1991) Studies on the biomass of Pinus yunnanensis forest. Acta Bot. Yunnanica 13, 59-64.

**Appendix 2.** Ecozone AGB (Mg $ha^{-1}$) - Stand age relationships for secondary forests.

| **Ecozone** | **Continent** | **Equation** | **Max age** |
|---|---|---|---|
| Tropical rainforest | Africa | AGB= -121.3 + {90.96 x ln(Age)} | 40 |
| | North and South America | AGB= -40.87 + {53.34 x ln(Age)} | 80 |
| | Asia | AGB= -12.56 + {27.17 x ln(Age)} | 40 |
| Tropical moist forest | Africa | AGB= -33.81 + {30.4 x ln(Age)} | 58 |
| | North and South America | AGB= -127.2 + {77.16 x ln(Age)} | 100 |
| | Asia* | AGB= -11.16 + {19.46 x ln(Age)} | 20 |
| Tropical dry forest | North and South America | AGB= -104.26 + {60.54 x ln(Age)} | 70 |
| Tropical mountain system* | Africa* | AGB= -113.1 + {74.14 x ln(Age)} | 63 |
| | North and South America | AGB= -72.03 + {53.65 x ln(Age)} | 50 |
| | Asia | AGB= -17.1 + {25.03 x ln(Age)} | 55 |
| Subtropical humid forest | Asia | AGB= -142.43 + {64.47 x ln(Age)} | 100 |
| Subtropical mountain system | Asia | AGB= -88.94 + {46.64 x ln(Age)} | 100 |

*Model only applicable for younger secondary forests.

**Appendix 3.** Number of chronosequences and permanent plots per 100,000 $km^2$ of natural forests in tropical and subtropical ecozones [a].

| Ecological Zone | Continent | No. of permanent plots | No. of chronosequences | No. of permanent plots and chronosequences per 100,000 $km^2$ of natural forests |
|---|---|---|---|---|
| Tropical rainforest | Africa | 105 | 16 | 4.68 |
| | North and South America | 324 | 42 | 6.20 |
| | Asia | 66 | 7 | 4.29 |
| Tropical moist forest | Africa | 2 | 2 | 0.21 |
| | North and South America | 27 | 21 | 3.07 |
| | Asia | 0 | 2 | 0.45 |
| Tropical dry forest | Africa | 0 | 5 | 0.39 |
| | North and South America | 1 | 7 | 0.82 |
| | Asia | 0 | 1 | 0.32 |
| Tropical shrublands | Africa | 0 | 1 | 0.26 |
| | North and South America | 0 | 0 | 0 |
| | Asia | 0 | 0 | 0 |
| Tropical mountain system | Africa | 0 | 3 | 1.05 |
| | North and South America | 6 | 6 | 1.82 |
| | Asia | 5 | 6 | 2.25 |
| Subtropical humid forest | Africa | 0 | 0 | 0.00 |
| | North and South America | 0 | 0 | 0.00 |
| | Asia | 0 | 24 | 5.14 |
| Subtropical dry forest | Africa | 0 | 0 | 0 |
| | North and South America | 0 | 0 | 0 |
| | Asia | 0 | 0 | 0 |
| Subtropical steppe | Africa | 0 | 0 | 0 |
| | North and South America | 0 | 0 | 0 |
| | Asia | 0 | 0 | 0 |
| Subtropical mountain system | Africa | 0 | 0 | 0 |
| | North and South America | 0 | 1 | 0.48 |
| | Asia | 0 | 32 | 5.85 |

[a] Extent of natural forests were obtained from Schulze *et al.* (2019) and combined with FAO (2012) to obtain coarse estimates of natural forest area per ecozone.
